# Injecting competition into online programming and Chinese- English translation classrooms

**DOI:** 10.3389/fpsyg.2024.1268734

**Published:** 2024-09-18

**Authors:** Yinjia Wan, Jian Lian, Yanan Zhou

**Affiliations:** ^1^School of Foreign Language Studies, Shandong Jiaotong University, Jinan, China; ^2^School of Intelligence Engineering, Shandong Management University, Jinan, China; ^3^School of Arts, Beijing Foreign Studies University, Beijing, China

**Keywords:** online education, engagement, academic performance, cooperative learning, intergroup competition

## Abstract

The introduction of competition has the potential to enhance the efficacy of students' learning performance. Nevertheless, there have been contradictory findings about the impact of intergroup competition on students' learning performance and engagement. Therefore, further comprehensive investigations for this problem are necessary. In order to bridge this gap, the present study seeks to ascertain the efficacy of intergroup competition in relation to students' academic performance and motivation. Consequently, we present the concept of intergroup competition and implement it within the context of an online programming course and an online Chinese-English translation course. The participants of this study consist of sophomore students majoring in Computer Science and English. Initially, a total of 108 sophomore students majoring in Computer Science participated. Then, a total of 100 sophomore students majoring in English participated. A quasi-experimental study was subsequently undertaken to compare students from two courses, which are online programming and Chinese-English translation, assigning them to an experimental group and a comparison group, respectively. Then, we conducted independent samples *t*-tests to measure the difference between the academic performance of the two group of students from two courses. The results indicate that both groups of students who were exposed to the intergroup competition mechanism demonstrated considerably higher levels of academic performance and engagement compared to the other group of students. The findings indicate that the competition mechanism, has the potential to be a beneficial instrument for enhancing both students' learning performance and motivation.

## 1 Introduction

Previous studies have regarded online learning as a valuable instrument for enhancing face-to-face learning activities (Albors-Garrigos et al., 2011; Arnal et al., 2017; English et al., 2022). In recent decades, Online learning has gained widespread acceptance as a viable educational strategy across different levels of education in recent decades (Adedoyin and Soykan, 2020; Almahasees et al., 2021; Alaali, 2022). This is primarily due to the potential of online learning approaches to facilitate accelerated and enhanced developmental trends (Campanella et al., 2008; Hodges et al., 2020; Tripon, 2022).

Numerous studies have been undertaken to investigate the implementation of intergroup competition inside university courses. In the work of Wood et al. (2005), it was observed that university students were able to learn course-related knowledge through their ability to adapt to competitive environments. In a study conducted by Lee et al. (2022) and Yu (2001), it was shown that fifth-grade kids exhibited improved learning outcomes after adopting intergroup competition. However, the implementation of intergroup competition as a cooperative method resulted in poorer performance in science class for students of the same grade. To note that current studies did not directly address the examination of intergroup competitive mechanisms within an online learning environment. Hence, further investigation needs to be conducted to explore the impact of intergroup competition in the context of online learning.

Despite several research have examined the various aspects affecting online learning, particularly in relation to online programming platforms, a significant gap remains in the literature about the omission of intergroup competitive mechanisms and their impact on these platforms. As elucidated in the aforementioned literature, a range of intergroup competition tactics can be utilized as a stimulating modality for fostering collaboration. These studies have also demonstrated their potential effectiveness in enhancing students' learning performance and engagement. Furthermore, the global emergence of the novel Coronavirus in 2020 has resulted in a reduction of the traditional offline learning period that typically coincides with online learning, potentially exerting a substantial impact on the process of online education. Hence, the purpose of this study was to investigate the impact of incorporating intergroup competition into an online platform on the academic performance and engagement of university students.

## 2 Literature review

Over the past few decades, significant advancements in information technologies have instigated transformative shifts within universities globally (Jones and O'Shea, 2004; Kurbel et al., 2009; Dwivedi et al., 2019; Liang and Cui, 2022). E-learning and the Internet are widely recognized as suitable platforms for delivering various sorts of courses to students, without spatial or temporal limitations. Numerous studies have been undertaken in the past to examine the impact of e-learning on university students (Yu and Yu, 2010; Mese and Sevilen, 2021). For instance, the study conducted by Chan and Waugh (2007) aimed to examine the various factors influencing the level of student engagement in the online learning environment (OLE) among mathematics distance learning students at the Open Learning University of Hong Kong (OUHK). Their objective was to identify potential recommendations for enhancing students' utilization of the OLE. A survey instrument was developed with the purpose of investigating the usage patterns of OLE among students. The results of the statistical analysis indicated that students had a positive inclination toward utilizing the OLE as a means of exchanging knowledge and engaging in collaborative learning. In addition, the students expressed a collective preference for receiving lesson notes and assignment solutions concurrently. The work of Liu et al. (2010) introduced the technology acceptance model as a fundamental framework and expanded upon the external and perceived variables inside their model. This study involved the participation of 436 senior high school students from Taiwan, who were engaged in an online learning community with a specific emphasis on English language acquisition. The research findings indicated that the inclusion of additional variables can be a reliable predictor of user adoption in an online learning community. In their study, Fan et al. (2021) examined the various factors that influence the motivation of online learners. The sample consisted of 93 participants, and the study considered components such as learners, educators, curriculum, and platform, as well as 13 subordinate factors. The findings of the study indicate that several factors, such as learning demand, self-efficacy, instructor personal traits, educational level, course material, course assessment, technical support, learning interaction, and incentive measures, have been observed to exert considerable positive influences on the motivation to engage in online learning. Nevertheless, there is a limited amount of research that has specifically examined the impact of competition mechanisms, which often include comparing one's performance to others who are completing the same activity (Coakley, 2007), on students' academic accomplishment and engagement. Interpretation of the social interdependence theory posits that intergroup competition emerges as a consequence of team members uniting to engage in competitive activities against competing groups. According to Deci et al. (1981), common consequences of engaging in competition encompass achieving victory and fostering a sense of collective pride within the group. Typically, the implementation of point-based rewards and leaderboards is a prevalent approach to stimulate competitiveness within various contexts (Chang et al., 2018; Hudja et al., 2020; Seaborn and Fels, 2015). One perspective suggests that the allocation of points by teachers or peers might serve as a direct source of motivation within an educational setting. On the contrary, leaderboards have their origins in the realm of cybergames and can be integrated into the online educational setting to augment students' motivation through the provision of immediate feedback. In the study conducted by Dreu et al. (2021), the authors investigated the impact of intergroup competition on the cooperative performance and interactive strategies of primary school children. A total of 80 students were selected to participate in a puzzle task, where they were divided into groups of four and instructed to collaborate. A total of eight groups were randomly allocated to the non-competitive condition, whereas twelve groups were placed to the competitive condition. The findings of this study indicated that intergroup competition had a suppressive effect on the frequency of communication among groups consisting of younger students, whereas it led to an expansion of communication among groups consisting of older students (Chen and Chiu, 2016). The present study devised a mechanism for intergroup competition and included it into a multi-touch platform designed for collaborative learning in the context of design-based education. The objective was to augment the engagement levels, learning outcomes, and creative abilities of primary school pupils. The results of the statistical study indicated that students who were exposed to intergroup competition had considerably higher levels of student engagement, learning accomplishment, and originality compared to students who were not exposed to competition.

Previously, many studies have examined the impact of competition on the academic performance of the students in middle schools. For instance, the work of Chan and Lam (2008) compared the influence of intergroup competition on students' writing self-efficacy in vicarious learning. In the competitive group, the self-efficacy decreased when the students engaged in vicarious learning. In the control group, the self-efficacy of students did not have a significant difference in vicarious learning. Furthermore, in a game-based learning context designed for the students of seventh-grade, the study Chen et al. (2019) revealed the impact of competition and engagement in games, as well as the associations between them on performance of the students in science learning. To note that this study did not conceive that competition alone had a direct effect on the students' performance. However, it was indirectly related to performance along with engagement. Then, in the study Chen et al. (2020), a meta-analysis was conducted to investigate the impacts of competition on digital game-based learning. From this research, it can be found that competition was effective in digital game-based learning for math, science and language except for social science. Meanwhile, competition was effective for K12 students and students in the universities. Ho et al. (2021) proposed a meta-analytical study, in which whether peer competition and peer collaboration moderated the effectiveness of gamification in learning performance was investigated. In their study, a moderating effect of peer competition in gamification in learning could be revealed, which suggested that competitive games were better than non-competitive games for promoting learning performance. Recently, Wang and Huang (2023) presented a question bank practice game to offer a situation of inter-group competition with intra-group collaboration. Meanwhile, a research model was devised to investigate the correlations between competition, collaboration, and learning performance. Its findings indicated that competition is a more significant factor than collaboration for learning performance.

It can be summarized that the current studies for revealing the influence of competition on students' performance are still limited to some extent. To be specific, the experts and scholars in this are have neglected the influence of competition on the students taking online courses, which needs to be studied in-depth.

### 2.1 Research questions

Bearing the above-mentioned analysis in mind, in this study we raised the following questions as:

- Is there a significant difference in academic performance between sophomore students who collaborate with their partners to complete a Python project or a Chinese-English translation project in online platforms, with the experiment group utilizing an intergroup competition mechanism, compared to the control group without such a mechanism?
- Does the introduction of an intergroup competition strategy, where sophomores collaborate with their partners to complete a Python project or a Chinese-English translation project in the same online platforms, result in higher levels of engagement for students in the experimental group compared to the control group without the intergroup competition mechanism?

## 3 Methodology

This research endeavored to delve into the intricacies of online learning dynamics by adopting a quasi-experimental design, which is a robust framework for comparing the experiences of two distinct groups—an experimental group subjected to a specific intervention and a control group that was not. The crux of this intervention was an independent variable, meticulously crafted to introduce a competitive element into the online learning environment. The study's primary focus rested on two pivotal dependent variables: academic performance and engagement. Academic performance was gauged through the traditional yet effective measure of testing results. These assessments were meticulously designed to evaluate the students' grasp of the course material and their ability to apply theoretical concepts in practical scenarios. The tests were administered at various intervals throughout the course to capture a comprehensive view of the students' learning trajectories. Engagement, on the other hand, was a more nuanced construct that required a different approach to measurement. To this end, a questionnaire was employed, crafted with care to encompass a wide array of factors that contribute to a student's active participation in the learning process. This included, but was not limited to, the frequency of logins, the depth of interaction with course materials, and the level of contribution to online discussions and group activities.

The initial stage of the study involved a thorough assessment of the students' learning performance through these testing results. This phase was critical in establishing a baseline from which the effects of the intervention could be measured. The results were meticulously analyzed to identify patterns, trends, and any potential outliers that could provide deeper insights into the learning process. To complement the quantitative data garnered from the tests, the qualitative data collected through the questionnaires offered a rich tapestry of information regarding the students' attitudes, motivations, and behaviors in the online learning environment. This dual-pronged approach allowed for a more holistic understanding of the impact of intergroup competition on learning outcomes.

For the statistical analysis, the study leveraged the capabilities of SPSS Statistics 22.0, a powerful tool renowned for its comprehensive suite of analytical features. This software facilitated the processing and interpretation of both the quantitative test scores and the qualitative questionnaire responses. The analysis was conducted with a keen eye for detail, ensuring that all data were accurately represented and that the findings were robust and reliable. To maintain the highest standards of scientific rigor, a two-tailed α threshold of 0.05 was employed for all statistical tests. This conservative approach to statistical significance ensured that any observed effects were not merely the result of chance, but rather indicative of a genuine impact of the intervention on the students' academic performance and engagement.

In summary, this study meticulously orchestrated a quasi-experimental design to explore the effects of intergroup competition on students' online learning. Through a combination of testing results and questionnaires, a comprehensive assessment of academic performance and engagement was conducted. The use of SPSS Statistics 22.0 and a stringent alpha threshold further underscored the study's commitment to producing meaningful, actionable insights into the complex interplay between competition and learning in the digital age.

### 3.1 Participants

For the online programming course, a total of 108 sophomore students, consisting of 48 females and 60 males, with ages ranging from 18 to 22 years [MEAN = 20.69, Standard Deviation (SD) = 1.62], who were majoring in computer science, software engineering, and information management at Shandong Normal University, were selected as participants. For the Chinese-English course, a total of 100 sophomore students, consisting of 72 females and 28 males, with ages ranging from 18 to 22 years (MEAN = 20.57, SD = 1.42), who were majoring in English at Shandong Normal University, were selected as participants. The students were enrolled in a series of courses for a duration of one year, which covered essential topics such as online programming and Chinese-English translation. During the Spring semesters of 2020 and 2022, a group of students enrolled in an online Python course and an online Chinese-English translation course, respectively. To note that they were unable to physically attend the in-person session as a result of the outbreak of the new Coronavirus. In this research, the participants were allocated to two separate groups, including the group adopting the intergroup competition mechanism and the group that did not adopt intergroup competition mechanism (or the control group).

### 3.2 Ethical approval

Ethical approval for this research was granted by the Institutional Review Board at Shandong Jiaotong University, reinforcing the study's commitment to ethical standards and academic integrity. Written informed consent was obtained for each participant according to institutional guidelines. The informed consent explains the study's purpose, the participants' rights, and the confidentiality of their responses.

### 3.3 Materials

A pre-test and a post-test were conducted to evaluate the difference between students who were exposed to the intergroup competition mechanism and those who were not. On the other hand, the previous assessment was employed to evaluate the students' foundational understanding of online programming and Chinese-English translation. Conversely, the post-test was administered as the culminating assessment for the online programming and Chinese-English translation. The students were mandated to finalize the examinations within a time frame of 60 min and 90 min, respectively.

### 3.4 Online programming and Chinese- English translation projects using intergroup competition mechanism

**Figure 1:**
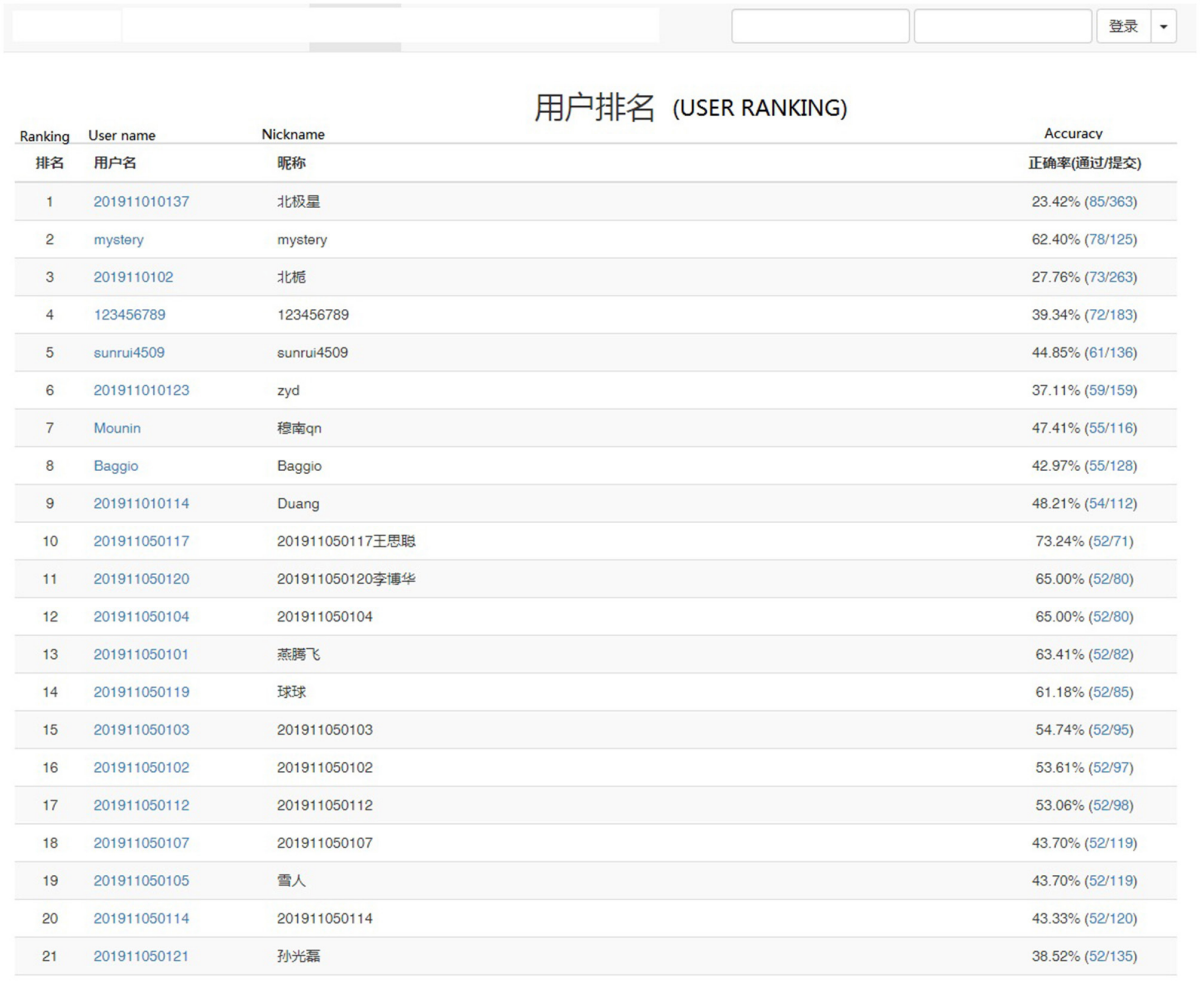
The leader-board in the online learning platform.

A group of three students was mandated to collaborate in order to develop a software application using the Python programming language. Prior to the commencement of this online learning course, we facilitated an online programming platform utilizing Visual C# 2015. This platform enabled students to effectively engage with their software development assignments. In order to evaluate the students' knowledge, it was necessary for them to undergo a preliminary examination. The evaluation process involved utilizing a standardized test paper with predetermined answers, which were graded accordingly. Throughout the duration of the 18-week course, a teacher and two assistant teachers evaluated each team by appraising the level of project completion and rating their performance in terms of teamwork on a weekly basis. In order to mitigate potential bias from the teachers, a method of ranking was employed wherein the average of three grades was utilized. Then, the students in each team were informed of their respective positions on the leaderboard, which was made available on the online learning platform (see to Figure 1). After the completion of the entire project, the students were instructed to partake in a post-test, wherein a test paper containing predetermined answers was utilized.

In a Chinese-English translation course, a team of three students was required to work together on a collaborative translation project. The online learning platform facilitated their engagement with the translation tasks effectively. To assess their proficiency, students were required to take a preliminary exam, which was conducted using a standardized test. Throughout the 18-week course, a teacher and two teaching assistants evaluated each team on a weekly basis. They assessed the completion level of the project and rated the students' teamwork. To ensure fairness and reduce teacher bias, the grading was done by averaging the scores from three different sources. The students were then informed of their standing on a leaderboard, which was accessible on the Chinese-English translation course's online platform. At the end of the project, students were asked to take a post-test, which used a test paper with predetermined correct answers.

In order to evaluate the level of student involvement and the impact of intergroup competition strategy on student engagement, a Chinese version of the UK involvement Survey (Bokhove and Muijs, 2019) was employed. The survey consists of 14 items designed to evaluate the quality of the learning materials, utilizing a 4-point Likert-type scale ranging from 1 (indicating minimal impact) to 4 (indicating significant impact).

## 4 Results

Two independent samples *t*-tests were conducted to evaluate the difference between the groups of students in the pre-test and post-test. As depicted in Tables 1, 2, the descriptive statistics encompassing the MEANs and SD for the dependent variables pertaining to both academic achievement and students' engagement are presented.

**Table 1:** MEANs and standard deviations of the dependent variables for the online programming course.

**Dependent variables**	**Competition group**		**Control group**	
	**MEAN**	**SD**	**MEAN**	**SD**
Academic performance	81.45	2.40	67.48	1.78
Engagement	47.20	2.55	39.10	2.20

**Table 2:** MEANs and standard deviations of the dependent variables for the Chinese-English translation course.

**Dependent variables**	**Competition group**		**Control group**	
	**MEAN**	**SD**	**MEAN**	**SD**
Academic performance	87.31	2.65	79.57	1.90
Engagement	52.40	2.46	44.60	2.76

### 4.1 Academic performance

The present study employed an analysis of covariance (ANCOVA) to examine the impact of students' programming or Chinese-English translation proficiency on their post-test performance. Specifically, the study aimed to compare the post-test scores of students in the competition group with those in the control group, while controlling for the influence of their prior test scores as a covariate. For the online programming course, a statistically significant distinction was observed between the competition group and the control group in terms of their academic performance, as indicated by the obtained *F*-value = 1.82 (*p*=0.02) and effect size (η_*p*_ = 0.96). Meanwhile, for the Chinese-English translation course, a statistically significant distinction was also observed between the competition group and the control group in terms of their academic performance with the *F*-value = 1.95 (*p* = 0.01) and effect size ($\eta_{p}^{2}=0.86$). For both courses, the post-test results revealed a considerable improvement in the performance of students in the competition environment compared to their counterparts in the control group.

### 4.2 Engagement

The researchers utilized an independent samples *t*-test to evaluate the difference in engagement levels between the competition group and the control group for both the online programming and Chinese-English translation courses. The findings from the online programming course of the *t*-test indicated a substantial difference in the level of student engagement [*t*_(106)_ = 17.67, *p* < 0.01, $\eta_{p}^{2}=0.86$]. The findings of the research indicate that the level of student engagement in the intergroup competition condition (MEAN = 47.20, SD = 2.55) was considerably higher compared to the control group (MEAN = 39.10, SD = 2.20). Meanwhile, the findings from the Chinese-English translation course of the *t*-test indicated a substantial difference in the level of student engagement [*t*_(98)_ = 14.92, *p* < 0.01, $\eta_{p}^{2}=0.83$]. And the level of student engagement in the intergroup competition condition (MEAN = 52.40, SD = 2.46) was also considerably higher compared to the control group (MEAN = 44.60, SD = 2.76).

## 5 Discussion

The study's findings, as outlined in Section 2.1, are remarkable. They clearly indicate that there are substantial differences in both academic performance and engagement between the students who were part of the inter-competition group and those in the control group. Moreover, the results suggest that students who engaged in intergroup competition achieved higher academic performance than their peers who did not participate in such competitive activities.

The results presented in the study conducted by Wood et al. (2005) support the notion that the implementation of intergroup competition might positively impact participants' learning performance and motivation. The findings of this study align with the research conducted by Tauer and Harackiewicz (2004), where they observed that the integration of competition and cooperation (specifically intergroup competition) consistently resulted in increased levels of intrinsic motivation. This study provides support for the research conducted by Roncarati et al. (2006), suggesting that inter-group competition, as opposed to collaboration, may enhance assessment performance and learning outcomes. The findings align with those of Akpinar et al. (2015), who observed that cultural variations in attitudes toward collaboration and competitiveness can potentially impact learning outcomes to some extent. The international competition and the opportunity to engage with real-life business problems serve as catalysts for students' active involvement and contribute to improved academic performance. The implementation of an intra-group collaboration framework, supplemented by an inter-group mechanism, has the potential to foster student motivation and active engagement in learning activities, while also facilitating positive social contact within the context of a design project. According to Chen and Chiu (2016), the practice of assigning points to students based on their behaviors is widely regarded as a potent motivator that has a positive impact on both their academic achievement and level of involvement. Consequently, it can be inferred that the students who were exposed to the intergroup competition condition would have achieved higher levels of academic performance compared to the students in the control group. In contrast, the study conducted by Yu (1998) investigated the comparative impacts of collaboration with and without inter-group competition on students' academic performance in science and their attitudes toward science within a Computer-assisted instruction (CAI) setting. The data that was acquired revealed notable disparities between the two situations in terms of student academic performance and student attitudes toward the field of science. Nevertheless, based on the analysis of the findings, it was recommended that the instructional strategy of intra-group cooperation without inter-group competition be used as the optimal approach. In contrast to the study conducted by Lam et al. (2001), which encompassed a broader range of dependent variables such as task enjoyment, achievement attribution, and test anxiety, our analysis focused solely on examining the impact of intergroup competition mechanism. Specifically, we evaluated this influence by measuring learning performance and engagement as the dependent variables. In the subsequent phase, we will proceed to incorporate additional dependent variables into the investigation. Recently, the work of Fernández Fernández et al. (2021) investigated the influence of transition from face-to-face teaching to online learning on students in college and the corresponding sustainability. It can be observed from the outcome of this study that despite being sustainable from an environmental, social, and economic perspective.

Despite the numerous discoveries from this study, it is crucial to acknowledge its limitations. First of all, the selection of the study population might have introduced subjectivity. To be specific, only the students majoring in Computer Science were selected partially because one of the researchers is majoring in Computer Science. In addition, due to the exclusive utilization of online learning and the absence of offline activities in this study, it is plausible that the implementation of this technique by classroom teachers may be limited. Hence, it is possible that the results obtained may not be applicable to the traditional face-to-face learning environment. Furthermore, it is important to note that this research was only carried out on a small group of sophomore students from one single university. Therefore, caution should be exercised when attempting to generalize the findings to a wider population encompassing other majors, grade levels, districts, and nations.

## 6 Conclusion

This study aimed to examine the effects of the intergroup competition mechanism on students' learning performance and engagement in the context of intra-group cooperation. This study represents an initial exploration of the online learning environment by integrating an intergroup competition mechanism into an online programming course and an online Chinese-English translation course. A quasi-experimental design was employed to compare the performance of students in the competition group with those in the control group.

From the research and literature review in this study, it can be observed that the introduced intergroup competition mechanism has proven its capability for enhancing student performance in online programming and Chinese-English translation contexts. Meanwhile, it can also be observed that competition is a potentially valuable instrument for improving the students' engagement. However, there are still several limitation of this study need to be acknowledged. First of all, the number of samples collected in this study is still limited, and more samples should be provided in our next studies. Moreover, since this is a case study, we did not take more influence factors into consideration, which might have neglected the impact of various factors on the students' performance and engagement.

In the future, to enhance the comprehensiveness of the analysis regarding the impact of the intergroup competition mechanism, it is recommended that further studies incorporate large number of data samples and more different types of dependent variables. In addition, we will also continue to study the other types of influencing factors.

## Data Availability

The raw data supporting the conclusions of this article will be made available by the authors, without undue reservation.
