# Corrigendum: Injecting competition into online programming and Chinese- English translation classrooms

**DOI:** 10.3389/fpsyg.2025.1561929

**Published:** 2025-06-03

**Authors:** Yinjia Wan, Jian Lian, Yanan Zhou

**Affiliations:** ^1^School of Foreign Language Studies, Shandong Jiaotong University, Jinan, China; ^2^School of Intelligence Engineering, Shandong Management University, Jinan, China; ^3^School of Arts, Beijing Foreign Studies University, Beijing, China

**Keywords:** online education, engagement, academic performance, cooperative learning, intergroup competition

In the published article, there was an error in the author list. The equal contribution symbol † was omitted for authors Jian Lian and Yinjia Wan. The symbol and the corresponding note “These authors have contributed equally to this work” have been added to the author list.

The authors apologize for this error and state that this does not change the scientific conclusions of the article in any way. The original article has been updated.

